# HIV-associated gut dysbiosis drives oncogenesis through metabolic-immune crosstalk: mechanisms and therapeutic implications

**DOI:** 10.3389/fonc.2025.1634388

**Published:** 2025-08-21

**Authors:** Qingquan Meng, Liran Xu, Furong Xu, Xiaohan Shen, Jingyu Yue

**Affiliations:** The First Affiliated Hospital of Henan University of Traditional Chinese Medicine, Zhengzhou, China

**Keywords:** HIV, gut microbiota, metabolite, tumor, immune

## Abstract

HIV-induced gut microbiota dysbiosis perpetuates mucosal barrier disruption and systemic inflammation despite antiretroviral therapy (ART), creating a tumor-permissive microenvironment. This review synthesizes evidence linking HIV-associated microbial alterations to oncogenesis through three convergent metabolic axes: (1) butyrate deficiency impairing epithelial energy metabolism and anti-tumor immunity; (2) tryptophan metabolism dysregulation compromising gut barrier integrity via *Akkermansia muciniphila* depletion and *Enterococcus*-mediated phenylethylamine overproduction; and (3) vitamin B biosynthesis defects disrupting DNA repair and Th1/Th2 balance. Comparative profiling across HIV-associated malignancies—non-Hodgkin lymphoma, cervical cancer, hepatocellular carcinoma, and lung cancer—reveals conserved dysbiotic signatures: depletion of anti-inflammatory taxa (*Bacteroidetes*, *Bifidobacterium*) and expansion of pro-inflammatory genera (*Proteobacteria*, *Shigella*). These alterations activate NF-κB/STAT3 signaling, fostering IL-6/TNF-α-driven chronic inflammation. Emerging interventions, including *Bifidobacterium*-derived metabolites and butyrate supplementation, demonstrate potential to enhance immunotherapy efficacy and reverse chemoresistance. However, causal microbiota-tumor relationships remain unproven, and key AIDS-defining cancers (Kaposi sarcoma, anal carcinoma) lack microbial association studies. Prioritizing longitudinal multi-omics analyses, organoid models, and LMIC-focused clinical trials may advance microbiota-directed strategies for HIV-associated cancer prevention and treatment.

## 1 Introduction

Acquired immunodeficiency syndrome (AIDS) is a chronic disease caused by the human immunodeficiency virus (HIV). As of 2023, global HIV/AIDS statistics reveal two critical challenges: an estimated 39.9 million people living with HIV/AIDS worldwide, and approximately 1.3 million new infections occurring in that year alone, underscoring its persistent status as a major global health crisis (1). Geographic disparities in disease burden are particularly striking, with sub-Saharan Africa accounting for nearly two-thirds of global HIV infections. Notably, over 90% of cases are concentrated in low- and middle-income countries (LMICs) (2), where vulnerable populations including women and children under 15 face heightened infection risks due to lower social demographic indices (3).

The health challenges in LMICs extend beyond HIV prevalence. These regions frequently experience synergistic health burdens including childhood stunting, malnutrition, environmental pollution, and enteric pathogen infections. Such conditions create a biological milieu where gut microbiota dysbiosis manifests more severe consequences than in high-income nations, predisposing populations to immune dysregulation, recurrent infections, and chronic inflammation (4). Compounding these challenges, LMICs often lack the advanced diagnostic technologies and healthcare infrastructure required for effective cancer management, resulting in disproportionate cancer-related morbidity (5). Emerging evidence suggests that gut microbiota dysbiosis may contribute to HIV-associated oncogenesis, but its causal role remains controversial. The key issues include bidirectional causality, the difficulty of predicting functional impacts through diversity metrics, and the direct alteration of the microbiome by HIV treatment regimens (6).

As the body’s central hub for digestive, immune, and metabolic functions, the gastrointestinal tract plays a pivotal role in maintaining physiological homeostasis. A healthy human gut hosts a complex ecosystem of over 100 trillion microorganisms, predominantly bacterial species (7). This microbiota fulfills essential physiological roles through: 1) establishing a biological barrier against pathogens; 2) enhancing intestinal development and metabolic capacity; 3) regulating nutrient absorption via interactions with intestinal epithelial and Paneth cells; 4) modulating immune cell proliferation and systemic immune maturation (8); 5) communicating with the brain via the gut-brain axis; and 6) aiding in the biotransformation and detoxification of harmful substances (6). Molecular signaling through bacterial components (lipopolysaccharides, peptidoglycans) and microbial metabolites (short-chain fatty acids [SCFAs]) further enables cross-regulation of immune cells including epithelial cells, macrophages, dendritic cells, and neutrophils (9–11).

Notably, the gastrointestinal tract serves as the primary site for HIV replication and pathogenesis. Viral infection triggers a cascade of pathological events: lymphocyte destruction, profound CD4+ T-cell depletion, pro-inflammatory cytokine storm, immune barrier disruption, and microbiota dysbiosis - all contributing to disease progression acceleration (12, 13). Crucially, even with successful viral suppression through highly active antiretroviral therapy (HAART), persistent defects remain in gut mucosal repair, microbial community structure restoration, and inflammatory resolution (14). These unresolved alterations constitute key barriers to immune reconstitution in treated patients (15).

Emerging therapeutic strategies targeting gut microbiota modulation show promise for addressing these challenges. Interventions including prebiotics, probiotics, fecal microbiota transplantation, and phage therapy demonstrate multifaceted benefits: 1) restoring microbial ecological balance and SCFA production (16); 2) enhancing gut barrier integrity (“leaky gut” repair); and 3) improving systemic immunity through increased CXCR3+ CD4+ T-cell populations and anti-inflammatory responses (17). Although some studies have reported that probiotics can reduce levels of inflammation markers such as D-dimer, clinical evidence supporting their role in promoting immune recovery in HIV-infected individuals remains limited. The heterogeneity in study design, including strain selection and intervention duration, may affect the evaluation of their efficacy (18).

HIV/AIDS-induced depletion of CD4+ T lymphocytes leads to progressive immunosuppression, creating a permissive microenvironment for tumorigenesis and metastatic progression (19). HIV-associated malignancies are clinically categorized into two distinct groups: AIDS-defining cancers (ADCs) and non-AIDS-defining cancers (NADCs) (20). The ADC classification encompasses Kaposi’s sarcoma, non-Hodgkin lymphoma, and invasive cervical carcinoma, while NADCs include anal carcinoma, hepatocellular carcinoma, and lung cancer (21, 22). Notably, emerging evidence implicates gut microbial dysbiosis in the pathogenesis of several HIV-associated malignancies, including non-Hodgkin lymphoma, cervical cancer, hepatocellular carcinoma, and lung cancer. For instance, in non-Hodgkin’s lymphoma, the microbiota metabolite phenylethylamine promotes abnormal B-cell proliferation and EBV activation (23). In HPV-related cervical and anal cancers, the reduction of Lactobacillus weakens local mucosal immunity, facilitating persistent HPV infection (24). In colorectal cancer, toxins from Fusobacterium nucleatum and Escherichia coli, such as colibactin, directly damage DNA and suppress the immune system (25). This growing body of research positions gut microbiota modulation as a promising therapeutic frontier for HIV-related oncogenesis.

Through systematic analysis of gut microbial alterations in HIV-associated malignancies (diffuse large B-cell lymphoma, cervical cancer, hepatocellular carcinoma, and lung cancer) integrated with metabolic network profiling (26), we identified three convergent pathological mechanisms: 1) Depletion of butyrate-producing bacteria disrupts epithelial energy metabolism and anti-tumor surveillance; 2) Microbial community shifts impair vitamin B biosynthesis, compromising DNA repair mechanisms; 3) Intestinal barrier dysfunction facilitates microbial translocation, triggering chronic systemic inflammation. These microbial-metabolic perturbations synergistically activate NF-κB and STAT3 signaling pathways, generating a pro-inflammatory cytokine milieu (IL-6, TNF-α, IL-1β) that establishes a tumor-promoting microenvironment through autocrine and paracrine cascades. Our tripartite framework - analyzing butyrate metabolism, intestinal barrier integrity, and vitamin B biosynthesis - elucidates critical intersections between HIV-induced gut dysbiosis and oncogenesis. This mechanistic stratification provides: (i) clinical biomarkers for monitoring malignancy risk in HIV patients, and (ii) therapeutic targets for developing microbiota-directed adjuvant therapies.

## 2 The HIV/AIDS gut microbiota

The gastrointestinal tract is the primary target for HIV pathogenesis, and is associated with alterations in the gut microbiota related to HIV/AIDS. Following HIV infection, characteristic microbiota dysbiosis manifests through three key alterations: 1) Quantitative reduction in microbial biomass and α-diversity; 2) Ecological shift marked by increased pathobionts (e.g., *Prevotella*, *Proteus*) and decreased beneficial taxa (e.g., *Lachnospira*, *Bifidobacterium*); 3) Functional niche displacement facilitating opportunistic pathogen expansion. Phylum-level analyses reveal significant depletion of Bacteroidetes concomitant with overrepresentation of Firmicutes, Proteobacteria, and *Clostridium* clusters, effectively compromising microbial antagonism and barrier protection (27, 28). Notably, specific microbial signatures correlate with disease progression. Li et al. identified *Shigella* enrichment and *Methylobacterium* depletion as biomarkers for severe CD4+ T-cell depletion (<200 cells/µL) (29). Longitudinal analysis by Zhang et al. demonstrated stage-dependent taxonomic shifts: Early HIV infection exhibits elevated *Enterococcus*, *Brevundimonas*, and *Aeromonas*, while advanced stages correlate with progressive loss of butyrogenic genera (*Faecalibacterium prausnitzii*, *Roseburia*) and expansion of pro-inflammatory taxa (*Enterococcus*, *Lactobacillus*) *(* 30). Antiretroviral therapy (ART) introduces additional microbial perturbations distinct from HIV-induced changes. Emerging evidence suggests certain ART regimens may exacerbate dysbiosis through microbiota composition alteration and enhanced microbial translocation (28). Post-ART stratification reveals persistent dysbiosis in both immune responders (IR) and non-responders (INR), characterized by depletion of immunomodulatory taxa (*Bifidobacterium*, *Eubacterium*) and enrichment of *Clostridium* clusters compared to healthy controls (31). The gut microbiota-mucosal barrier axis plays pivotal roles in HIV pathogenesis. Dysbiosis-induced mucolytic activity reduction, particularly the depletion of *Akkermansia muciniphila* (“gut sentinel”), disrupts critical homeostatic mechanisms: 1) Impaired mucus layer maintenance through diminished epithelial mucin secretion (32); 2) Compromised cross-feeding networks for butyrate producers (*F. prausnitzii*, *Roseburia*) that lack autonomous mucin degradation capacity (33). The microbiota influences the progression of HIV and HPV infections through immune regulation, metabolic intervention, and mucosal barrier modulation (show in **Table 1**).

**Table 1:** Gut microbiota dysbiosis in HIV/AIDS and various tumors.

Disease	Bacteria that decreases in number	Bacteria that increases in number	References
HIV/AIDS	Phylum: Bacteroidetes; Genus: F. prausnitzii, Lachnospira, Ruminococcaceae_UCG-002, Roseburia, Dorea, Alistipes, Bifidobacterium, Eubacterium, Roseburia, Methylobacterium, Akkermansia muciniphila	Phylum: Firmicutes, Proteobacteria; Genus: Proteus, Shigella, Prevotella, Clostridium, Enterococcus, Brevunmdimonas, Aeromonas, Pseudomonas, Lactobacillus, Ruminococcus, Megamonas	(27–32)
Non-Hodgkin’s lymphoma (DLBCL)	Phylum: Bacteroidetes; Genus: Allisonella, Lachnospira, Roseburiain	Phylum: Proteobacteria, p_Verrucomicrobia; Class: γ-Proteobacteria, c_Verrucomicrobiae、c_Bacilli; Order: Enterobacterales, o_Verrucomicrobiales; Family: Enterobacteriaceae, Genus: Shigella, Enterococcus, Veillonella, Prevotella-2, g_Acidaminococcus, g_Akkermansia, g_Pyramidobacter, g_Streptococcus	(39, 40)
Cervical cancer	Genus: Bacteroides, Alistipe, Lachnospira, Ruminococcus-2	Family: Porphyromonadaceae, Enterobacteriaceae; Genus: Prevotella, Porphyromonas, Dialister, Shigella, Enterobacter	(45–47)
Hepatocellular carcinoma	Phylum: Actinobacteria; Family: Bifidobacteriaceae, Clostridiaceae, Bacteroidaceae, Lachnospiraceae, Rikenellaceae, Christensenellaceae, Peptostreptococcaceae, Coriobacteriaceae, Prevotelaceae, Oscillospiraceae; Genus: Bifidobacterium, Ruminococcus	Class: γ-Proteobacteria; Family: Enterococcaceae, Enterobacteriaceae, Suttrellaceae, Streptocochaceae; Genus: Enterococcus, Proteus, Klebsiella	(51, 52)
Lung cancer (NSCLC)	Phylum: Firmicutes, Actinobacteria, Bacteroidetes; Genus: F. prausnitzii, Veillonella, Streptococcus, Bifidobacterium, Clostridium UCG-14	Phylum: Proteobacteria; Family: Lachnospiraceae, Coriobacteriaceae, Rikenellaceae; Genus: Ruminococcus, *Blautia obeum, Akkermansia muciniphila*, Lactobacillus, Intestinimonas, Salmonella, Turicibacter, Eisenbergiella, DTU089, Ruminococcaceae Incertae Sedis; Species: *Lactobacillus salivariu*	(56–58)

## 3 Gut microbiota profiling in HIV-associated malignancies

### 3.1 HIV-associated non-Hodgkin lymphoma

HIV-associated lymphomagenesis arises from multifactorial interactions involving virus-specific mechanisms and microbial dysregulation. The pathogenic triad of HIV-induced immunosuppression, direct viral oncogenesis, and gamma herpesvirus co-infections (EBV/HHV-8) creates a permissive microenvironment for lymphoma development (34). Clinical epidemiology demonstrates striking correlations between immunosuppression severity and lymphoma risk, with CD4+ T-cell counts below 200 cells/µL and elevated HIV viral load serving as independent risk predictors. Notably, HIV-positive non-Hodgkin lymphoma (NHL) patients exhibit doubled mortality rates compared to HIV-negative counterparts within two years post-diagnosis (59% *vs*. 30%) (35). Emerging evidence positions gut microbiota dysbiosis as a fourth pathogenic dimension through dual mechanisms of chronic inflammation potentiation and immune checkpoint dysregulation, directly associating microbial imbalance with increased NHL risk (36). Among NHL subtypes, diffuse large B-cell lymphoma (DLBCL) and Burkitt lymphoma (BL) predominate in HIV-infected populations, with DLBCL showing particularly strong gut microbiota associations (37, 38).

Comparative microbiota analyses reveal distinct ecological shifts in untreated DLBCL patients. Yuan et al. demonstrated through 16S rRNA sequencing of 25 DLBCL patients and 26 controls a characteristic dysbiosis pattern featuring Proteobacteria expansion (particularly γ-Proteobacteria/Enterobacteriaceae) and Bacteroidetes depletion at phylum levels. Genus-level alterations included pathogenic enrichment (*Shigella*, *Enterococcus*) alongside commensal depletion (*Allisonella*, *Lachnospira*). Mechanistically, *Escherichia coli*-derived genotoxins (colibactin/CDT) induce epithelial DNA damage through double-strand break formation, while Proteobacteria overgrowth correlates with therapy resistance and immune checkpoint inhibition (39). Lin et al. further identified progressive dysbiosis in advanced DLBCL through 16S rDNA analysis of 35 patients, showing stage-dependent increases in *Verrucomicrobia*, *Bacilli*, and *Streptococcus* despite preserved α/β diversity. Notably, Bacteroidetes abundance positively correlated with butyrate production capacity, suggesting metabolic consequences of phylum-level shifts (40).

The conserved microbial signature shared between HIV infection and DLBCL pathogenesis encompasses Proteobacteria/*Prevotella* expansion coupled with Bacteroidetes/*Lachnospira* depletion. This overlapping dysbiosis profile suggests a continuum of microbial-immune interactions contributing to lymphomagenesis, where enterococcal overgrowth and butyrogenic deficit may synergistically drive inflammation-mediated oncogenesis.

### 3.2 HIV-associated cervical cancer microbiota

Cervical cancer (CC) represents the most prevalent AIDS-defining malignancy, with HIV-infected women exhibiting six-fold higher incidence compared to the general population (41). This elevated risk stems from synergistic interactions between HIV and human papillomavirus (HPV), particularly pronounced in sub-Saharan Africa where HIV contributes to 20% of regional CC burden (42). The co-pathogenesis mechanism involves: 1) HIV-mediated impairment of HPV clearance; 2) Increased susceptibility to high-risk HPV subtypes (43); and 3) Accelerated progression from cervical intraepithelial neoplasia (CIN3) to invasive carcinoma, even under antiretroviral therapy (44). Emerging evidence implicates gut microbiota dysbiosis as a co-factor through estrogen metabolism modulation and systemic immunosuppression, facilitating HPV persistence and carcinogenesis (45).

Comparative microbiota profiling of 42 CC patients versus 46 controls revealed three consistent alterations: 1) Enrichment of pro-inflammatory genera (*Prevotella*, *Porphyromonas*); 2) Depletion of immunoregulatory taxa (*Bacteroides*, *Alistipes*); and 3) *Lachnospira* deficiency impairing mucosal homeostasis. Mechanistic studies demonstrate *Legionella pneumophila* activates TLR2-mediated IL-17/Th17 axis via dendritic cell stimulation (IL-1β/IL-6/IL-23), establishing chronic pro-carcinogenic inflammation (46).

Radiotherapy-induced microbiota changes show temporal dynamics: Early-stage chemoradiotherapy (CRT) patients exhibit *Porphyromonas* dominance, while long-term survivors develop *Shigella*/*Enterobacteriaceae* enrichment (47). Crucially, enhanced microbiota diversity correlates with improved survival outcomes, mediated through activated CD4+ T-cell infiltration and optimized anti-tumor immunity. Clinical parameter correlations further validate: 1) Inverse associations between patient age and *Bacteroides* abundance; 2) Disease stage progression with *Shigella* overgrowth; and 3) Protective roles of *Ruminococcus 2* in neoplasia suppression (45). The shared microbial signature between HIV and CC pathogenesis features *Prevotella*/*Shigella* expansion coupled with *Bacteroides*/*Lachnospira* depletion, suggesting conserved pathways of dysbiosis-driven carcinogenesis in AIDS-related malignancies.

### 3.3 HIV-associated hepatocellular carcinoma microbiota

The epidemiological landscape of HIV-associated malignancies has shifted with antiretroviral therapy (ART) success: Reduced AIDS-defining tumor incidence contrasts with rising non-AIDS-defining cancers, particularly hepatocellular carcinoma (HCC). This transition reflects ART-mediated viral suppression, immune reconstitution, and prolonged survival, unmasking HCC as a growing oncological burden in aging HIV populations (48). Coinfection dynamics play pivotal roles, as shared transmission routes between HIV and hepatotropic viruses (HBV/HCV) amplify HCC risk. HIV-infected individuals exhibit a higher HBV/HCV prevalence and an elevated HCC incidence versus HIV-negative counterparts, with a lower CD4+ T-cell counts cells/µL further enhancing risk (49). Gut-liver axis dysregulation emerges as a central pathogenic mechanism. Microbial metabolites translocate via the portal vein, impairing hepatic immunosurveillance through: 1) Disrupted tight junction integrity from short-chain fatty acid (SCFA) depletion; 2) Enterococcus-driven lipopolysaccharide (LPS) leakage activating proinflammatory cascades; and 3) Treg cell dysregulation via butyrate deficit (50). Zhang et al. conducted longitudinal analysis of 74 male HCC patients, revealing progressive microbiota alterations: Early-stage disease showed enrichment of Lachnospiraceae and Peptostreptococcaceae, while terminal stages exhibited marked Enterococcaceae/Enterobacteriaceae expansion alongside depletion of Bifidobacteriaceae and SCFA-producing taxa (51). Yan et al. extended these findings through comparative analysis of 90 subjects (HBV-HCC, HBV-liver cirrhosis, healthy controls), identifying conserved dysbiosis patterns in chronic liver disease: Proinflammatory γ-Proteobacteria and Streptococcus enrichment coupled with reduced butyrate producers (*Ruminococcus*, *Oscillospira*) *(* 52). This microbial configuration suggests gut-derived immunomodulation significantly influences hepatocarcinogenesis. The convergent microbiota signature in HIV and HCC patients features: pathobiont predominance (*Enterococcus*/Proteobacteria), commensal depletion (*Bifidobacterium*/Lachnospiraceae) and bacteroidaceae family reduction. These alterations establish a pathogenic cycle of barrier dysfunction, microbial translocation, and hepatic inflammation, underscoring the gut microbiota’s role in HIV-associated HCC progression.

### 3.4 HIV-associated lung cancer microbiota

Lung cancer (LC) constitutes a predominant non-AIDS-defining malignancy and leading cause of mortality among HIV-associated tumors. While elevated smoking rates in HIV-infected populations contribute significantly to LC etiology, emerging evidence identifies independent HIV-specific risk factors including uncontrolled viral load, CD4+ T-lymphocyte depletion, recurrent bacterial pneumonia, and accelerated aging processes (53). Notably, HIV infection demonstrates an independent association with LC development beyond conventional risk factors (54). The compromised gut mucosal barrier in HIV infection facilitates microbial translocation, perpetuating systemic inflammation and immunosuppression even under antiretroviral therapy (ART). This dysbiosis-driven pathophysiology may mediate pulmonary carcinogenesis through bacterial dissemination, inflammatory cascade activation, and metabolic perturbation (54, 55). Zheng et al. conducted 16S rRNA sequencing in 42 early-stage LC patients and 65 healthy controls, revealing phylum-level dysbiosis characterized by Bacteroidetes/Proteobacteria expansion and Firmicutes/Actinomycetota depletion, potentially impairing short-chain fatty acid (SCFA) production. Genus-level analysis identified *Ruminococcus* enrichment alongside *Faecalibacterium prausnitzii* and *Bifidobacterium* reduction in early LC. Metastatic cases uniquely exhibited *Akkermansia muciniphila* and *Blautia obeum* predominance. Concurrent metabolic pathway alterations (steroid biosynthesis, bile secretion) in LC-associated microbiota suggest systemic pathophysiological modulation (56). Non-small cell lung cancer (NSCLC), representing the majority of LC cases, demonstrates distinct microbial signatures. Tesolato et al. reported decreased Bacteroidetes and increased *Lactobacillus*/*Salmonella* in 19 NSCLC patients versus 20 controls, with *DTU089* and *Ruminococcaceae Incertae Sedis* emerging as NSCLC-specific biomarkers (57). Therapeutic implications surface from Grenda et al., analyzing 214 advanced NSCLC patients receiving immune checkpoint inhibitors (ICIs): Antibiotic pretreatment correlated with *Bifidobacteriaceae* depletion and reduced ICI efficacy, while *Butyricicoccaceae* abundance predicted first-line therapy response. *Akkermansia muciniphila* enrichment associated with favorable treatment outcomes, and Firmicutes predominance correlated with prolonged progression-free survival (58). The overlapping gut microbiota profile between HIV infection and LC progression features Proteobacteria/*Ruminococcus* expansion and Bacteroidetes/*Bifidobacterium* depletion, with post-treatment *Akkermansia* resurgence suggesting conserved microbial remodeling patterns. However, mechanistic elucidation of gut-lung axis interactions in HIV-associated pulmonary carcinogenesis requires further investigation.

## 4 Metabolic regulatory networks of dysbiosis in HIV/AIDS gut microbiota

**Figure 1:**
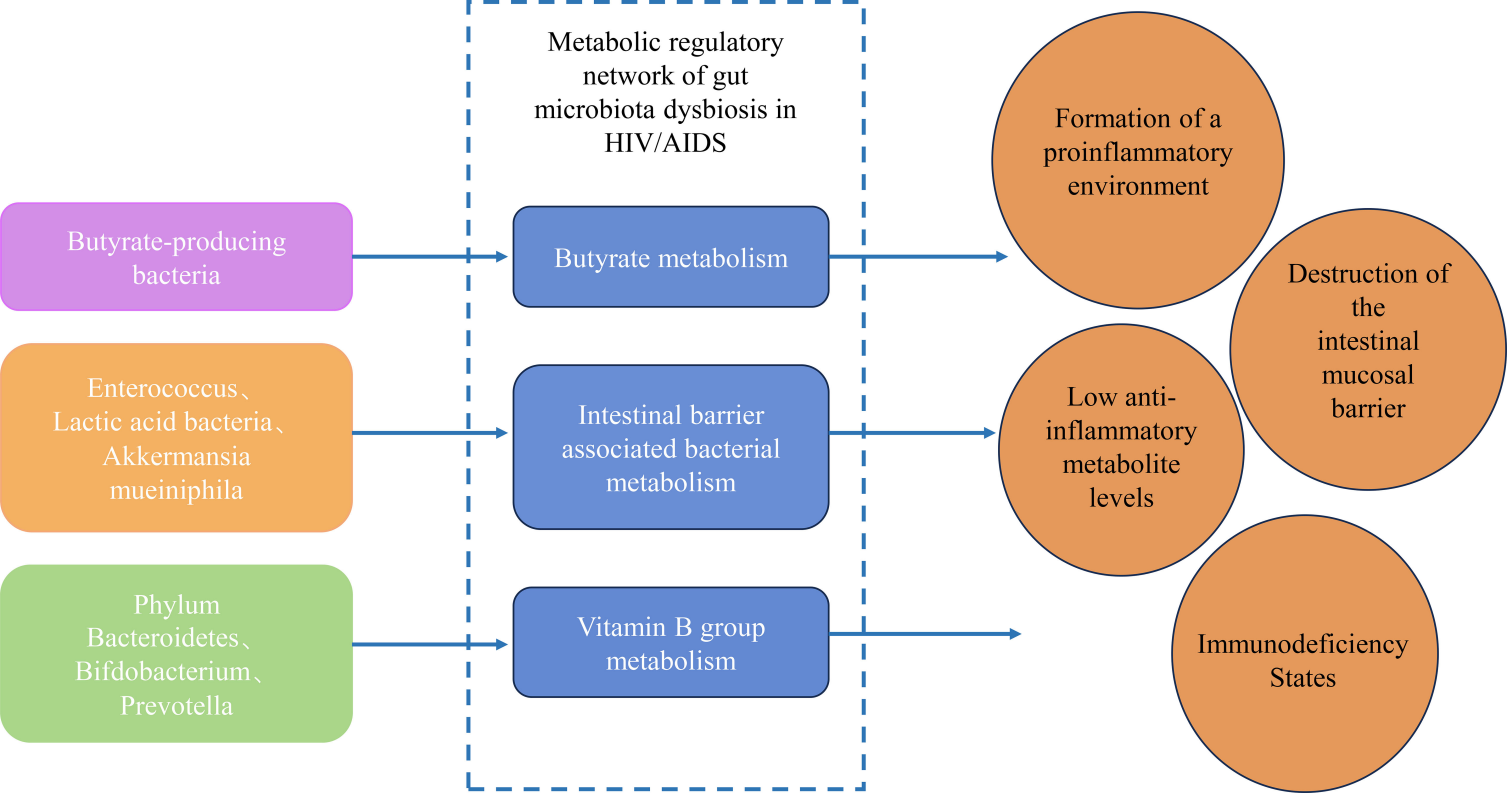
Metabolic regulatory network of gut microbiota dysbiosis in HIV/AIDS.

The gut microbiota in HIV/AIDS undergoes profound compositional and functional shifts, disrupting key metabolic pathways that govern immune homeostasis, barrier integrity, and anti-tumor surveillance. These perturbations arise from the depletion of symbiotic taxa critical for nutrient synthesis and the expansion of pathobionts that exacerbate inflammation. Central to this dysregulation are three interconnected metabolic axes: butyrate biosynthesis, gut barrier-associated bacterial metabolism, and vitamin B-dependent immunomodulation. Each axis contributes to a vicious cycle of microbial translocation, chronic inflammation, and immune exhaustion, creating a permissive milieu for oncogenesis (**Figure 1**).

### 4.1 Butyrate metabolism

HIV-associated gut dysbiosis significantly reduces butyrate-producing strains including *Faecalibacterium prausnostzii*, *Lachnospira*, and *Roseburia*, impairing butyrate biosynthesis (30). Butyrate originates primarily from microbial fermentation of undigested carbohydrates via glycolysis (EMP), pentose phosphate (HMP), and Enter-Doudoroff (ED) pathways, with additional production through acetate/lactate cross-feeding (59). Intestinal absorption occurs via H+-coupled (MCT1) and Na+-coupled (SMCT1) monocarboxylate transporters (49) (**Supplementary Figure S1**). This microbial metabolite exerts pleiotropic effects through three principal mechanisms:

Butyrate has immunomodulatory, anti-inflammatory and anti-tumor effects (60). Butyrate enhances regulatory B cell (Breg) immunosuppression by upregulating 5-hydroxyindole-3-acetic acid (5-HIAA) via *Bifidobacterium*-mediated tryptophan metabolism, activating aryl hydrocarbon receptor (AhR) and IL-10 signaling (61). Butyrate can also promote the proliferation and activation of regulatory T cells (Treg cells) and plays an important role in immune regulation (62). Additionally, butyrate can modulate the activity of inflammation-related pathways such as G protein-coupled receptors (GPRs), NF-κB, and JAK/STAT, thereby reducing the release of pro-inflammatory cytokines, inhibiting intestinal inflammatory responses, and maintaining intestinal immune balance (63). Concurrently, it induces dendritic cell (DC) expression of IDO1 and Aldh1A2 through histone deacetylase inhibition (HDACi), promoting FoxP3+ Treg differentiation while suppressing IFN-γ+ proinflammatory T cells (64). Butyrate stabilizes intestinal barrier function via HIF-1 activation in epithelial cells (65) and modulates NF-κB signaling to suppress TNF-α/IL-6 production (66). G-protein coupled receptor activation (GPR41/FFAR3, GPR109A/HCAR2) further enhances mucosal immunity through IgA secretion during inflammation (67).

In terms of anti-tumor activity, normal gut epithelial cells derive energy primarily from butyrate β-oxidation, whereas tumor cells rely on glycolytic metabolism—a metabolic shift termed the “Warburg effect” that supports rapid proliferation in hypoxic microenvironments (68, 69). Exploiting the Warburg effect, butyrate accumulates in tumor cells due to their preferential glycolysis over oxidative metabolism. This HDAC inhibition induces chromatin remodeling, cell cycle arrest, and apoptosis - mechanisms demonstrated to overcome sorafenib resistance in hepatocellular carcinoma (70). It is hypothesized that HIV-induced butyrate deficiency establishes a pathogenic triad of intestinal barrier disruption, chronic inflammation, and immune dysfunction, creating a permissive microenvironment for tumorigenesis and metastasis.

### 4.2 Gut barrier-associated bacterial metabolism

HIV-associated gut dysbiosis is characterized by enrichment of *Enterococcus* and lactic acid bacteria (*Lactobacillus*) alongside depletion of *Akkermansia muciniphila* (Akk), collectively contributing to mucosal barrier compromise and microbial translocation. This altered microbial consortium demonstrates enhanced L-tryptophan metabolic activity, with *Enterococcus* and *Lactobacillus* abundance positively correlating with intestinal tryptophan levels (71) (**Supplementary Figure S2**). Tryptophan catabolism proceeds through three primary routes: (i) IDO1-mediated conversion to kynurenine (KP) in immune/gut epithelial cells, suppressing TH17 differentiation and reducing IL-17/IL-22 production to impair mucosal repair (72); (ii) bacterial tryptophanase-dependent generation of AhR/PXR-activating indole derivatives modulating barrier integrity (73) and (iii) chromaffin cell serotonin synthesis via tryptophan hydroxylase 1 (TPH1), the rate-limiting enzyme catalyzing tryptophan hydroxylation to 5-hydroxytryptophan (5-HTP), which is subsequently decarboxylated to form 5-HT (74).

*Enterococcus* expansion in HIV infection drives phenylethylamine overproduction, directly inducing epithelial shedding (30) and potentiating microbial translocation (75). Concurrent akkermansia depletion may potentially disrupt critical protective mechanisms. One possible mechanism involves TLR2-mediated AMPK activation, which could enhance tight junction assembly (ZO-1/claudin-2) through the regulation of CDX2. Additionally, inhibition of NF-κB might lead to a reduction in pro-inflammatory cytokines (such as TNF-α and IL-6), while potentially elevating anti-inflammatory mediators like TGF-β and IL-10 (76) (**Supplementary Figure S3**). Furthermore, it is hypothesized that Akkermansia’s metabolic symbiosis with butyrate producers may be compromised by the loss of mucin-derived acetate/propionate and nutrient supply (30, 77). These alterations—potential overgrowth of pathobionts and depletion of sentinel species—may contribute to a microenvironment conducive to tumorigenesis, potentially through sustained barrier dysfunction and dysregulation of inflammatory-immune responses.

### 4.3 Vitamin B metabolism

HIV-associated gut dysbiosis disrupts vitamin B metabolism, critically impairing anti-inflammatory and immunoregulatory functions. Vitamin B1 (thiamine) serves as an essential cofactor in glycolysis, TCA cycle, and pentose phosphate pathways (78). *Bacteroides* species synthesize thiamine pyrophosphate (TPP) through dedicated transporters (79), with HIV-induced *Bacteroides* depletion correlating with reduced Peyer’s patch B-cell follicle development and compromised intestinal immunity (80). Vitamin B3 (niacin/nicotinamide) modulates redox balance via NAD metabolism (81), where deficiency associates with decreased gut microbiota α-diversity and *Bacteroidetes* depletion (82). Niacin exerts anti-inflammatory effects through PGD2/DP1-mediated vascular stabilization and IL-8 suppression (83), while restoring LPS/IL-1β-induced metabolic disturbances in isoleucine and glutamine pathways (84). Vitamin B6 exists as six vitamers, with pyridoxal phosphate (PLP) constituting the bioactive form. Gut *Bacteroides fragilis* and *Bifidobacterium longum* mediate its synthesis, while deficiency promotes *Prevotella* expansion (79). B6 deficiency disrupts Th1/Th2 balance, reducing IL-2 while elevating IL-4/IL-5/IL-10, impairing T-cell immunity (85). PLP additionally functions as a kynurenine pathway cofactor with tumor-suppressive potential (86). Clinical evidence from 44 HCC and 28 decompensated cirrhosis patients demonstrates impaired hepatic B6 metabolism correlating with disease progression (87). Murine models reveal vitamin B6 supplementation alleviates CCl4-induced hepatocyte damage and inflammation, while pyridoxine (PN) preserves intestinal barrier integrity by inhibiting advanced glycation end products (88). In non-HIV systems, depletion of Bacteroidetes/Bifidobacterium and Prevotella enrichment correlate with vitamin B deficiency states that foster pro-inflammatory microenvironments. In the context of HIV, synergistic dysbiosis may potentially amplify such deficiencies, creating a hypothesized metabolic-immune milieu conducive to tumorigenesis. However, direct evidence linking HIV-specific vitamin B deficiency to oncogenesis remains limited and warrants mechanistic validation.

## 5 Discussion

The gastrointestinal tract constitutes HIV’s primary reservoir, sustaining viral replication and progressive mucosal damage despite antiretroviral therapy (ART). Persistent gut microbiota dysbiosis and unresolved epithelial barrier defects perpetuate systemic immune activation and chronic inflammation, establishing a tumor-permissive microenvironment characterized by immunosuppression and metabolic dysregulation. Among HIV-associated malignancies, non-Hodgkin lymphoma (DLBCL), cervical carcinoma, hepatocellular carcinoma (HCC), and non-small cell lung cancer (NSCLC) exhibit conserved gut microbial signatures: depletion of anti-inflammatory taxa (*Bacteroidetes*, *Bifidobacterium*, butyrate producers) alongside expansion of pathogenic genera (*Proteobacteria*, *Shigella*, *Enterococcus*). These alterations disrupt microbial antagonism and niche protection, mirroring dysbiosis patterns observed in HIV infection.

Metabolic network analysis reveals three interconnected pathways linking HIV-induced dysbiosis to oncogenesis: (i) butyrate deficiency impairing epithelial energy metabolism and anti-tumor surveillance; (ii) tryptophan metabolism dysregulation compromising gut barrier integrity; (iii) vitamin B biosynthesis defects disrupting DNA repair and immune homeostasis. Synergistically, these perturbations activate NF-κB/STAT3 signaling, generating a pro-inflammatory cytokine milieu (IL-6, TNF-α, IL-1β) that promotes tumor progression through autocrine-paracrine cascades.

Emerging microbiota-directed interventions demonstrate therapeutic potential. *Bifidobacterium pseudolongum*, through its metabolite acetate, activates the GPR43 receptor and inhibits the activity of the IL-6/STAT3 signaling pathway via this mechanism. The IL-6/STAT3 pathway plays a role in promoting tumor growth and metastasis in various cancers, and thus its inhibition may slow the progression of HCC (89). PD-1/PD-L1 inhibitors are important tools in current cancer immunotherapy, capable of restoring the immune system’s attack on cancer cells by preventing tumor immune evasion. The co-administration of probiotics can modulate the gut microbiota, thereby enhancing the efficacy of PD-1/PD-L1 inhibitors and boosting the function of immune cells within the gut, significantly prolonging the survival of NSCLC patients (90). Although HCC and NSCLC share certain commonalities in tumorigenesis and immune regulation mechanisms, such as inflammation-driven tumorigenesis and immune evasion, they also exhibit distinct differences in specific molecular mechanisms. Further research is needed to elucidate the specific roles of these mechanisms. Butyrate supplementation reverses cisplatin resistance in cervical cancer by inhibiting epithelial-mesenchymal transition (91). Despite these advances, clinical translation requires rigorous validation through multicenter trials.

Collectively, HIV-associated gut dysbiosis fosters tumorigenesis through tripartite mechanisms: immunodeficiency potentiation, mucosal barrier disruption, and metabolic-immune crosstalk dysregulation. The conserved microbial and metabolic signatures across malignancies provide actionable biomarkers for cancer risk stratification and therapeutic targeting in HIV-associated oncogenesis.

## 6 Future perspective

This review delineates conserved microbial signatures across HIV infection and associated malignancies, revealing fundamental interconnections between gut dysbiosis and oncogenesis. Our analysis discusses three cardinal microbial-metabolic disturbances driving tumor progression in immunocompromised hosts: butyrate biosynthesis collapse, intestinal barrier disintegration, and vitamin B metabolism disruption. These pathological alterations synergistically activate NF-κB/STAT3 signaling cascades, generating a self-perpetuating inflammatory milieu characterized by IL-6/TNF-α/IL-1β overproduction that facilitates tumor immune evasion and metastatic progression. The convergent depletion of immunomodulatory taxa (*Bacteroidetes*, *Bifidobacterium*) alongside expansion of pro-inflammatory genera (*Proteobacteria*, *Enterococcus*) establishes a microbial profile predictive of cancer risk in HIV patients, transcending specific malignancy types.

Emerging therapeutic strategies targeting microbial metabolites demonstrate clinical potential yet require rigorous validation. Probiotic interventions and metabolite supplementation show capacity to restore gut-liver axis homeostasis in HCC, enhance checkpoint inhibitor efficacy in NSCLC, and reverse chemotherapy resistance in cervical carcinoma. However, critical knowledge gaps persist regarding causal microbiota-tumor relationships and temporal dynamics of dysbiosis progression. Current limitations include insufficient longitudinal cohort data tracking microbial changes pre-/post-malignancy development, and inadequate mechanistic models validating metabolite-mediated oncogenic pathways. Notably, two AIDS-defining cancers (Kaposi sarcoma, anal carcinoma) lack established microbiota associations, warranting dedicated investigation to determine potential gut-virome interactions.

Future research directions should prioritize: 1) Multi-omics longitudinal studies correlating microbial shifts with tumorigenesis timelines; 2) Organoid models elucidating metabolite-mediated epigenetic regulation; 3) Clinical trials evaluating microbiota modulation as adjuvant therapy. Particular emphasis should address LMIC-specific challenges including enteric pathogen co-infections and nutritional deficiencies that exacerbate HIV-related dysbiosis. By resolving these scientific and translational barriers, we may develop microbiota-based biomarkers for early cancer detection and personalized therapeutic regimens tailored to HIV-associated oncogenesis mechanisms.

## Supplementary material

Supplementary Figure 1 Butyrate metabolism.

Supplementary Figure 2 Tryptophan metabolism.

Supplementary Figure 3 Akkermansia muciniphila biological function.
