# Supplementary figures and images for: HIV-associated gut dysbiosis drives oncogenesis through metabolic-immune crosstalk: mechanisms and therapeutic implications
Source: Front Oncol. 2025 Aug 21;15:1634388. doi: 10.3389/fonc.2025.1634388 (PMC12408271; doi:10.3389/fonc.2025.1634388)

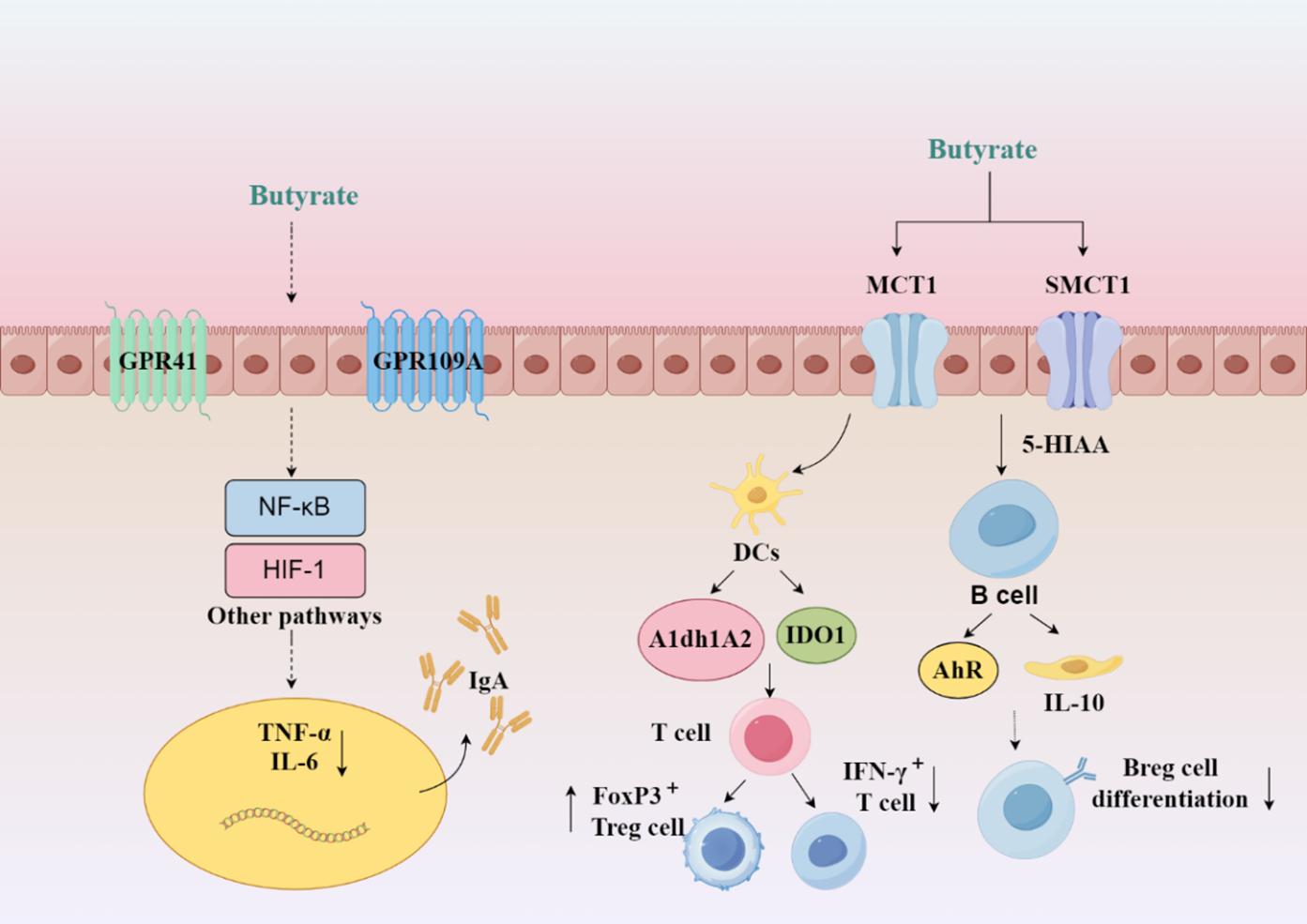

Supplement: Supplementary Figure 1 — Butyrate metabolism. [file Image1.png]

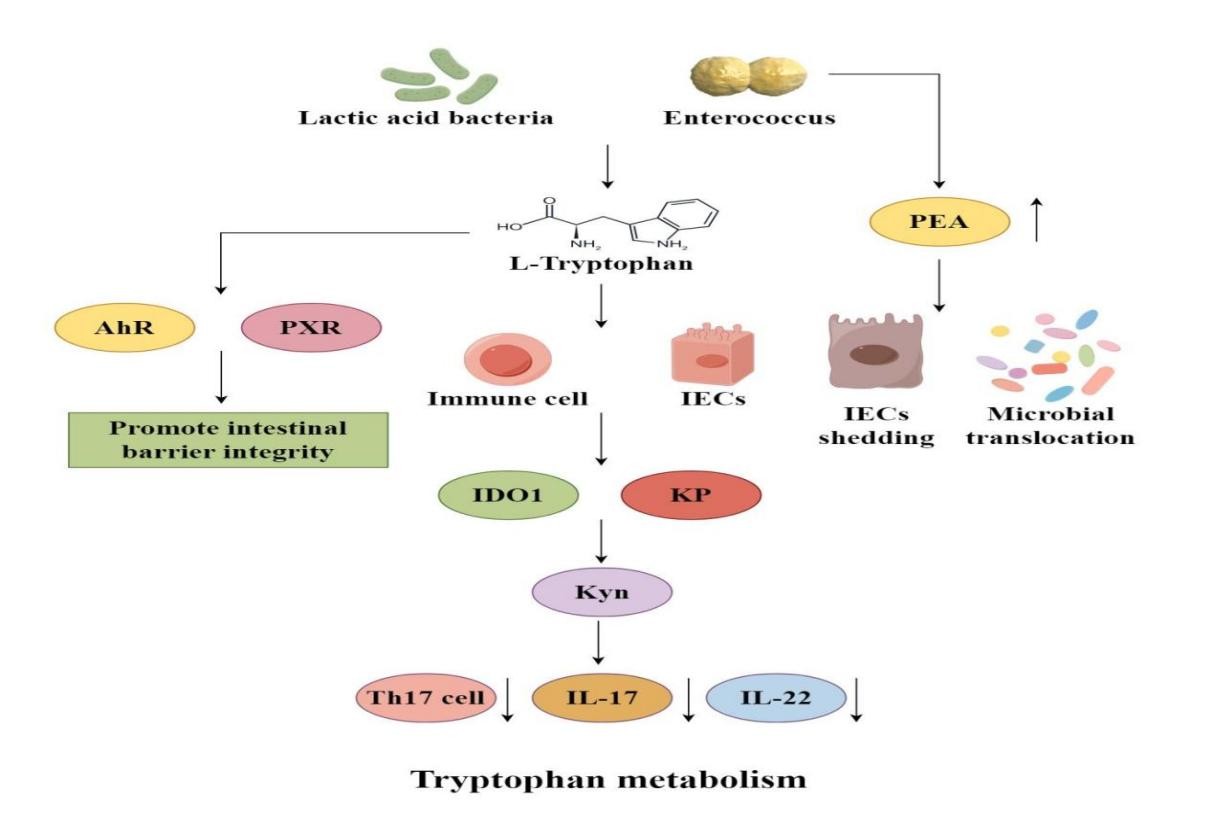

Supplement: Supplementary Figure 2 — Tryptophan metabolism. [file Image2.jpeg]

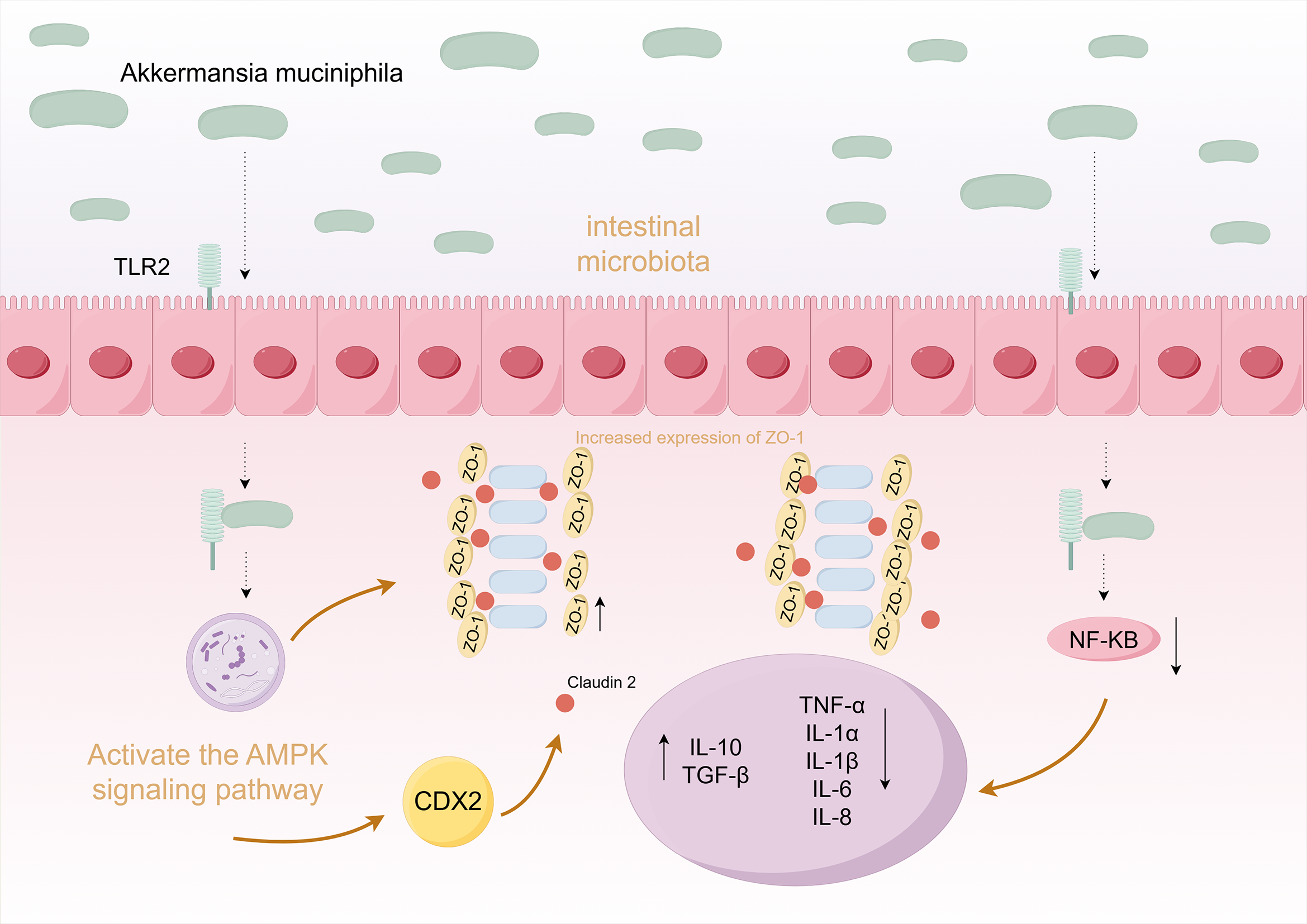

Supplement: Supplementary Figure 3 — Akkermansia muciniphila biological function. [file Image3.tif]
